# Integrated care model and point of care diagnostics facilitate Hepatitis C treatment among patients receiving opioid agonist therapy: a retrospective review of medical records

**DOI:** 10.1186/s13011-022-00473-3

**Published:** 2022-06-02

**Authors:** Margareeta Häkkinen, Jouni Tourunen, Tuuli Pitkänen, Kaarlo Simojoki, Sauli Vuoti

**Affiliations:** 1 A-Clinic Ltd, Ratamestarinkatu 7a, FI-00520 Helsinki, Finland; 2 Finnish Institute for Health and Welfare, P.O. Box 30, FI-00271 Helsinki, Finland; 3 A-Clinic Foundation, Ratamestarinkatu 7a, FI-00520 Helsinki, Finland; 4 Finnish Youth Research Society, Kumpulantie 3, FI-00520 Helsinki, Finland; 5 Department of Psychiatry, University of Helsinki, P.O 22, FI-00014 Helsinki, Finland; 6 Department of Clinical and Pharmaceutical Chemistry, University of Jyväskylä, P.O. Box 35, FI-40014 Jyväskylä, Finland

**Keywords:** Hepatitis C, People who inject drugs, Opioid agonist therapy, Linkage to care, Point of care diagnostics

## Abstract

**Background:**

Hepatitis C virus (HCV) is common among individuals in opioid agonist therapy (OAT). HCV treatment has previously been unavailable for most HCV positive OAT patients in Finland. The removal of treatment restrictions and attempts to reach HCV elimination goals have increased the number of OAT patients needing HCV treatment. The objectives of this study were 1) to characterize Finnish HCV positive OAT patients and evaluate their eligibility for HCV treatment at addiction service units, and 2) to retrospectively review the outcomes of treated patients.

**Methods:**

The study focused on HCV positive OAT patients (*n* = 235). Demographics and clinical parameters were retrospectively reviewed using the patients’ medical records. The eligibility of providing HCV treatment to patients at addiction service units were evaluated based on patients’ clinical characteristics, such as liver function and patterns of substance use. The outcomes of patients receiving HCV treatment were reviewed.

**Results:**

Of HCV antibody positive OAT patients, 75% had chronic HCV. Of 103 HCV patients screened for liver fibrosis either with Fibroscan or APRI (aspartate aminotransferase to platelet ratio index), 83 patients (81%) had no indication of severe liver damage. Point of care (POC) HCV tests were used for 46 patients to lower the threshold of attending laboratory testing. All patients preferred POC testing to conventional blood testing.

Twenty patients had received HCV treatment, 19 completed the treatment and achieved sustained virologic response (SVR) at the end of the treatment. Of the 18 patients available for evaluation of SVR at 12 weeks after the treatment (SVR12), 17 achieved SVR12.

**Conclusions:**

The integrated model consisting of HCV diagnostics and treatment at the addiction service unit was successfully implemented within normal OAT practice.

## Background

One of the World Health Organization’s (WHO) global strategy is to eliminate hepatitis C virus (HCV) infections by 2030 [1]. To reach this goal, it is essential to reach all populations at risk for HCV with testing and treatment. This study is in line with the WHO targets of decentralizing and expanding HCV services, and developing models of integrated and linked service delivery.

The availability of simple and tolerable interferon-free direct-acting antiviral agents (DAAs) for chronic HCV infection represents one of the most notable advances in clinical medicine in the past several decades. Adherence and response to DAA therapy among people who inject drugs receiving opioid agonist therapy (OAT) in clinical trials are viewed as comparable to those of populations without a history of injecting drugs [2]. OAT can increase linkage to HCV treatment [3]. Global treatment recommendations also encourage treating patients in OAT [4]. Recent studies demonstrate the feasibility of upscaling DAA therapy in high-risk people who inject drugs, and the therapy has shown to have potential individual and population-level public health benefits [2, 5, 6]. According to these studies, adequate information, support and counselling provided to this group as well as integrating HCV treatment into OAT, health counselling or primary health care, are crucial factors increasing motivation to seek treatment among individuals.

Finland’s Hepatitis C Strategy initiative has estimated that in 2019, the number of persons infected with HCV was over 30,000 out of 5.5 million inhabitants [7]. By the end of 2019, 32,939 HCV antibody positive cases had been entered into the Finnish National Infectious Diseases Register since the establishment of the register in 1998 [8]. In Finland, around 1150 persons are infected each year, and the disease burden is slowly increasing because of the amount of treatment provided has fallen behind as the number of persons requiring treatment has increased [9]. A global review concluded that 80% of high-income countries are not on track to meet HCV elimination targets by 2030, and 67% are off track by at least 20 years [10]. Immediate action to improve HCV screening and treatment globally has been suggested as a measure to eliminate HCV worldwide.

People who inject drugs are at risk of HCV infection [11]. While drug use has previously prevented the provision of HCV treatment, this restriction was lifted in 2018. A national strategy [7] and national recommendation on the cascade of care [9] determined that all persons should receive testing and treatment regardless of their drug user status. People who use drugs have previously been left systematically untested in Finland, which has led to the current nationwide estimates based on regional pilot programs. Before these pilot programs, HCV treatment has been available at departments of gastroenterology. Based on current estimates, at least 60% of the HCV-positive population in Finland are drug users [9].

Studies have shown that the initiation of HCV treatment among OAT patients is typically prevented by, for instance, a lack of disease symptoms, anticipated side effects from the treatment, and the presence of comorbid conditions and previous negative experiences from healthcare [12, 13]. Models focusing on interdisciplinarity, availability and accessibility through decentralized clinics and frequent follow-ups have been suggested to be effective [14–16].

A program aimed at integrating HCV treatment into standard OAT services by creating a realistic and adjustable treatment model adaptable for any OAT service provider was implemented in Finland in 2018. The integrated HCV treatment model aimed at including HCV testing, treatment, and follow-up in the OAT program. Evaluation of the integrated treatment model included initial treatment of twenty HCV positive patients. The objectives of the present retrospective review were 1) to investigate the clinical characteristics of HCV positive OAT patients in an aim to evaluate whether treatment could be administered at a addiction service unit, and 2) to summarize the HCV treatment and OAT outcomes of 20 patients who initially received HCV treatment.

## Methods

### Design

This study was a retrospective review of medical records. The patients’ electronic medical records were examined for laboratory and imaging data, demographic data, and follow-up data covering the period between October 1st, 2017, and December 31st, 2019. The data were retrieved from an electronic patient information system which consists of natural data entered into the system by professionals during the OAT process. Laboratory nurses had entered blood test data in the electronic patient record system. Addiction clinic nurses had collected demographic and concomitant drug use data based on patient interviews.

The study protocol has been approved by the A-Clinic Foundation’s Ethical Committee for Treatment and Research, and by the Finnish Institute for Health and Welfare. Consent was not required from patients for the use of this anonymized retrospective register data for research purposes.

### Setting

The data were extracted from three outpatient addiction service units located in Espoo, Helsinki, and Kouvola, and an inpatient unit of the A-Clinic Ltd’s Addiction Hospital, located in Järvenpää, Finland. The Addiction Hospital offers nationwide detoxification and rehabilitation for patients with substance use disorders, including OAT patients. A-Clinic Ltd. is owned by the A-Clinic Foundation, a non-profit, non-governmental organization, which provides services to municipalities under local agreements and does not provide services without a contract.

### HCV positive OAT patients

To investigate the clinical characteristics of HCV positive OAT patients, the reviewed data included all HCV antibody positive OAT patients (*n* = 235) who had voluntarily participated in HCV-related imaging or laboratory testing at their addiction service unit or the Addiction Hospital. HCV-RNA (ribonucleic acid) tests and genotyping were offered for all the HCV antibody positive patients in Espoo and Helsinki during spring 2018. HCV-RNA tests without genotyping were offered for all patients at the Addiction Hospital during a one-week period in autumn 2018 and a one-week period in spring 2019.

The liver functionality of the HCV positive OAT patients was defined using aspartate aminotransferase to platelet ratio index (APRI) or Fibroscan. A proportion of patients with an APRI score of <1 or Fibroscan score F0-F1 was defined to evaluate eligibility for HCV treatment at the addiction service unit without a need to refer the patients to specialized hospital care. APRI was measured at the Espoo and Helsinki addiction service units and the Addiction Hospital for the purpose of preparing the OAT patients for HCV treatment. Fibroscan measurements were offered for all the voluntary OAT patients in Espoo, Helsinki, Kouvola, and the Addiction Hospital during a 1–2-week period in autumn 2018 at all the study sites, and, additionally, during a two-week period in spring 2019 at the Addiction Hospital. In addition, some patients’ liver function had been evaluated in specialized hospital care and related data were included in their medical records. Trained addiction clinic nurses performed Fibroscan measurements, and physicians interpreted the results.

### Comparative databases

Each year, the Finnish Institute for Health and Welfare (THL) in Finland conducts a separate survey to collect data on clients seeking substance abuse services due to drug use or abuse of pharmaceutical compounds [17]. As part of the survey, each individual seeking these services fills out one questionnaire once per year. The aim of this national data collection is to provide comprehensive data on problematic drug use and drug treatment in Finland. The results are published annually in a THL report describing individuals using substance abuse services, and as a part of Finland’s national reporting to the European Monitoring Centre for Drugs and Drug Addiction (EMCDDA). The data are also used for scientific research and for reference at addiction service units. In our review, we compared the collected data with the aforementioned THL national data set from 2018 [18]. In our data, demographic variables including gender, age, education, employment and housing status, OAT medication, and data of concomitant drug use had been recorded following the treatment demand indicator (TDI) protocol, which enabled comparison with the national THL data [17].

Two other data sets were used for comparing the scope and intensity of psychosocial functioning: 269 OAT patients at the state-funded addiction service units maintained by the City of Helsinki [19], and 1082 inpatients hospitalized in the Addiction Hospital between 2014 and 2017 [20]. The patients were asked to fill out the PARADISE24fin questionnaire that includes 24 questions on common psychosocial difficulties rated using a scale from 0 (no difficulties/problems) to 4 (extreme difficulties/cannot do). The PARADISE24 method is evidence-based [21] and the items have been linked to the International Classification of Functioning, Disability and Health (ICF) [22].

### HCV treatment protocol and evaluation

Twenty out of the identified 235 HCV antigen positive OAT patients had received HCV treatment at the addiction service units in Espoo and Helsinki. Treatment inclusion criteria were HCV-RNA positive status and genotype 1, no serious insufficiency of liver (APRI<1), no renal impairment, no pregnancy or lactation, compatibility of DAA medication with OAT and other medication, and voluntary participation and motivation to adhere to the HCV treatment process. At the time of treatment, only a treatment for genotype 1 was reimbursed. According to Finnish national recommendation on the cascade of HCV care, patients with APRI ≥1 should receive their HCV treatment at the specialized health care instead of addiction service units to ensure patient safety in case of advanced liver disease.

The patients were initially enrolled in a training session on HCV that involved discussing with an addiction service nurse and the physician in charge of the patient’s treatment. HCV treatment was determined by the treating physician according to national recommendations. All therapies were given at European Medicines Agency (EMA) approved doses. HCV treatment and support were managed by a multidisciplinary care team comprised of an addiction medicine specialist, addiction service nurse, and an infectious disease physician providing remote consultation. This remote consultation included approval of the HCV treatment protocol and inclusion and exclusion criteria, and evaluation of the patients’ other medications and possible interactions with HCV medications. Illicit drug use was not an exclusion criterion. The patients were, however, required to control their drug use to the extent that they were able to visit the clinic regularly and take the medication daily according to plan. The patients were required to use contraception during the HCV treatment, and female patients were tested for pregnancy before starting the HCV medication.

HCV treatment was delivered simultaneously with the administration of OAT medication either at the clinic or with the patient’s home dosage according to each patient’s individual OAT treatment plan. The maximum duration of administering OAT medication at the patient’s home at a time was 6 days; therefore, the patients visited the clinic at least once a week. The patients were advised to take the HCV medicine orally once per day. Patients who had an institutional treatment period during the HCV treatment received their HCV medicine daily at the institution. Psychosocial support was offered in two ways: both group meetings and personal discussions with the care personnel were available. The treatment protocol included four group meetings, including peer support and information on HCV, HCV treatment, and avoiding a new infection after the treatment. Additional physician’s or nurse’s appointment were available according to request.

The addiction service nurses collected patient data on OAT and the treatment progress, including on injecting and concomitant drug use, and by interviewing the patients, performing urine drug tests, and making observations during regular clinical follow-ups. Follow-up data were collected on the patients’ OAT process regularly, 1–4 times per year according to the patients’ individual treatment plans. These medical records were qualitatively reviewed 3 months and one year after the HCV treatment to assess the outcomes of the HCV treatment and OAT performance.

The outcomes of the HCV treatment included completing the treatment, EOT (end of treatment) SVR, and SVR12 (SVR at 12 weeks after the treatment). OAT performance outcomes included the assessment of the amount of concomitant drug use and injecting drugs before and after the treatment, and psychosocial difficulties using PARADISE24fin [21].

### POC (point-of-care) testing

Besides using standard venous blood tests, HCV can be detected using point-of-care (POC) diagnostic methods. Of the POC methods, a dry blood spot (DBS) test is especially valuable for patients, as it provides an opportunity for HCV screening, diagnosis, and treatment with a low financial threshold without a need to purchase expensive equipment [23, 24]. The use of POC methods aimed to increase the proportion of patients taking laboratory tests at their own initiative by removing the requirement to refer the patient to an external laboratory, and in addition to alleviating sample extraction from patients with a history of injecting drugs and collapsed veins. Initiating all blood sample extraction at a addiction service unit may decrease the risk for dropouts due to patients refusing to, or being incapable of, visiting external laboratories.

The Xpert HCV Viral Load (Cepheid, Sunnyvale, CA) provided a quantitative HCV RNA result. Capillary blood was collected by trained personnel following an established procedure [25], using Accu-Check Safety Safe-T-Pro Plus lancets (Roche, Burgess Hill, UK) and Microvette 100 K3 EDTA collection tubes (Sarstedt, Leicester, UK). In addition, the dried blood spot (DBS) analytical method, as reported by [26], was used for diagnostics. DBS testing was offered for all the patients at the Espoo OAT clinic in spring and autumn 2018. POC testing was performed for voluntary OAT patients at the Addiction Hospital during a one-month period in spring 2019. We used these data as the basis for estimating the proportion of technical difficulties in venous puncturing among HCV positive OAT patients and collected the patients’ qualitative assessments of POC testing experiences.

### Statistical analysis

Demographic, OAT performance and psychosocial data were presented with frequencies, and percentages and their 95% confidence intervals. In statistical comparisons, we used the Pearson’s chi-square test for categorical variables, and the Mann-Whitney U test for continuous variables. A p value of <0.05 was considered statistically significant. The tool for statistical analysis was SPSS 26.0.

## Results

### HCV data

**Fig. 1:**
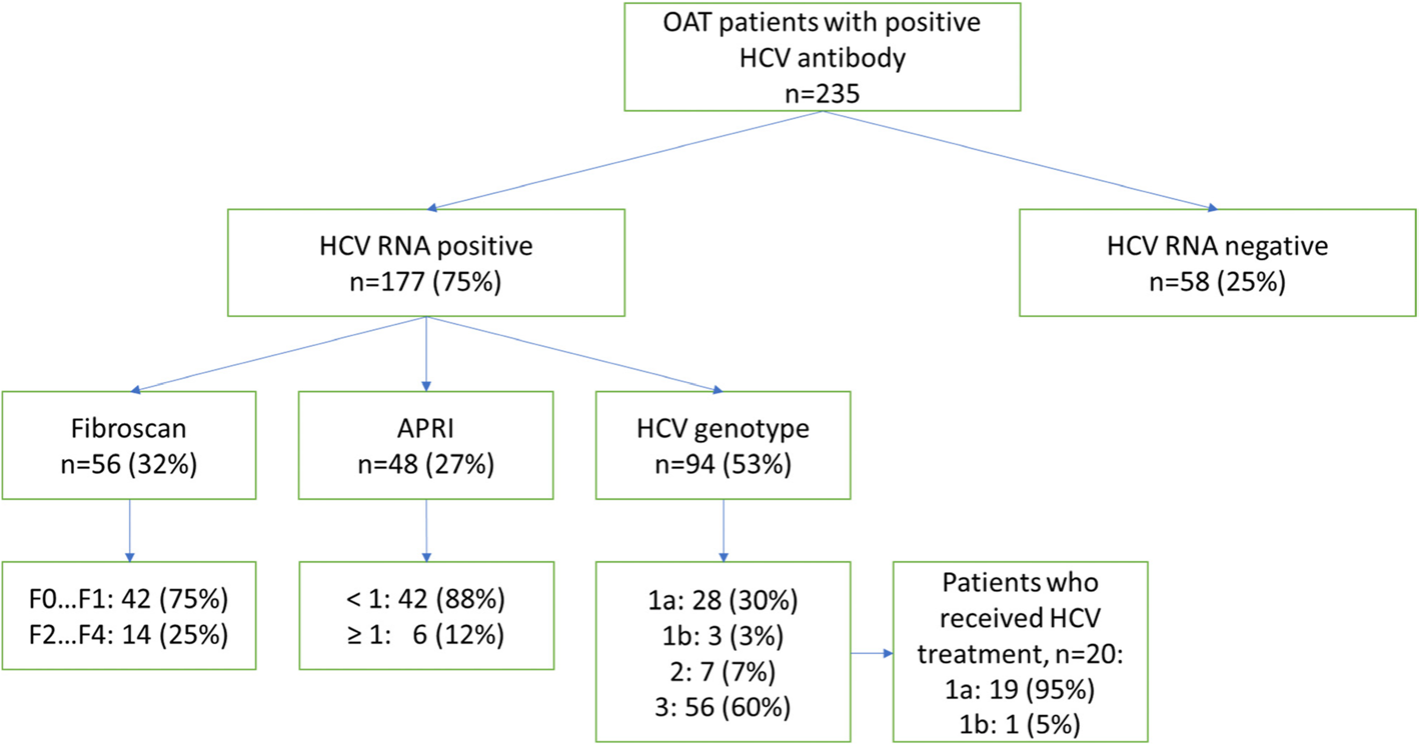
HCV laboratory and imaging data. OAT opioid agonist therapy, HCV hepatitis C virus, RNA ribonucleic acid, APRI aspartate aminotransferase to platelet ratio index. Fibroscan liver fibrosis stages: F0 … F1: no or mild fibrosis, F2 … F4: stages from moderate fibrosis to cirrhosis

Of the 235 HCV antibody positive patients identified in our review based on the patients’ medical records, 75% were HCV RNA positive and 25% had experienced a spontaneous recovery (Fig. 1). Of the total of 103 patients screened for liver fibrosis either with Fibroscan or APRI, 83 patients (81%) had no indication of severe liver damage defined as APRI >1 or Fibroscan >F1. One patient was screened for both APRI and Fibroscan, and both of the results indicated no liver fibrosis.

### Demographic and OAT data

The demographic and OAT-related data of the HCV-treated patients and the other HCV antibody positive OAT patients resembled each other, even though all the patients in the HCV treatment group were outpatients (Table 1). Also, the characteristics of HCV antibody positive OAT patients and the OAT treatment group resembled those of the OAT patients in the national data used as a comparison group [18]. The studied HCV positive patients were, however, somewhat more likely to have regular housing and to be in outpatient treatment than the OAT patients in the national data. The studied HCV positive patients reported more concomitant substance use, but less concomitant opioid use.

**Table 1:** Demographic and OAT data of the HCV treatment group and other HCV antibody positive OAT patients compared to each other and to the national OAT data [18]

	Data from patient records			Comparative data
	HCV treatment group (*n* = 20) % [95% CI]	Other HCV antibody positive OAT patients (*n* = 215) % [95% CI]	*P* value^a^	National OAT data (*n* = 836) [18] % (95% CI)
**Gender**	***n*** **= 20**	***n*** **= 215**	0.751	***n*** **= 836**
Male	65.0 [44.1,85.9]	67.9 [61.7,74.1]		71 [68,74]
**Age**	***n*** **= 20**	***n*** **= 215**	0.083	***n*** **= 836**
Median (min, max)	40 years (min 23, max 51)	38 years (min 20, max 59)		36 years (NA)
Under 30 years	10.0 [0.0,23.1]	14.9 [10.1,19.7]		20 [17,23]
30-39 years	25.0 [6.0,44.0]	46.0 [39.3,52.7]		45 [42,48]
40 years and older	65.0 [44.1,85.9]	39.0 [32.5,45.5]		35 [32,38]
**Education**	***n*** **= 20**	***n*** **= 177**	0.469	***n*** **= 836**
9 years or less	65.0 [44.1,85.9]	59.9 [52.7,67.1]		61 [58,64]
More than 9 years	30.0 [9.9,50.1]	40.1 [32.9,47.3]		33 [30,36]
Unknown	5.0 [0.0,14.6]	0.0		6 [4,8]
**Housing**	***n*** **= 20**	***n*** **= 178**	0.452	***n*** **= 836**
Regular	95.0 [85.4100]	88.8 [84.2,93.4]		81 [78,83.7]
Irregular	5.0 [0.0,14.6]	3.9 [1.1,6.7]		17 [14,20]
Homeless	0.0	7.3 [3.5,11.1]		0
Other or unknown	0.0	0.0		2 [1,3]
**Working status**	***n*** **= 20**	***n*** **= 178**	0.591	***n*** **= 836**
Regular	10.0 [0,23.1]	5.1 [1.9,8.3]		6 [4,8]
Irregular	0.0	3.4 [0.7,6.0]		1 [0,2]
Rehabilitative work activities	15.0 [0.0,30.7]	9.6 [5.3,13.9]		8 [6,10]
Unemployed	45.0 [23.2,66.8]	60.7 [53.5,67.9]		55 [52,58]
Retired	10.0 [0.0,23.1]	5.1 [1.9,8.3]		10 [8,12]
Other or unknown	20.0 [2.4,37.5]	16.3 [10.9,21.7]		20 [17,23]
**OAT treatment setting**	***n*** **= 20**	***n*** **= 194**	0.021	***n*** **= 836**
Inpatient	0.0	24.7 [18.6,30.8]		40 [37,43]
Outpatient	100.0	75.3 [69.2,81.4]		57 [54,60]
Unknown	0.0	0.0		3 [2,4]
**Concomitant substance use**	***n*** **= 20**	***n*** **= 179**		***n*** **= 836**
Current use	90.0 [76.9100]	71.5 [64.9,78.1]	0.083	55 [52,58]
Current IV/IM use	40.0 [18.5,61.5]	48.6 [41.3,55.9]	0.778	41 [38,44]
Stimulants	50.0 [28.1,71.9]	38.5 [31.4,45.6]	0.918	50 [47,53]
Opioids	5.0 [0.0,14.6]	16.2 [10.8,21.6]	0.109	46 [43,49]
Other drugs^b^	80.0 [62.5,97.5]	55.9 [48.6,63.2]	0.111	NA
Alcohol	15.0 [0.0,30.7]	16.8 [11.3,22.3]	0.603	30 [27,33]
**OAT program**	***n*** **= 20**	***n*** **= 169**	0.898	***n*** **= 836**
Rehabilitative	55.0 [33.2,76.8]	59.8 [52.6,67.0]		47 [44,50]
Harm-reducing	45.0 [23.2,66.8]	40.2 [33.0,47.4]		45 [42,48]
Undefined or unknown	0.0	0.0		8 [6,10]
**Years in OAT**	***n*** **= 19**	***n*** **= 194**	0.983	
median (min … max)	3 (min 0, max 17)	3 (min 0, max 18)		NA
**OAT medications (%)**	***n*** **= 20**	***n*** **= 203**	0.084	***n*** **= 836**
Methadone	45.0 [23.2,66.8]	51.7 [44.8,58.6]		45 [42,48]
Buprenorphine-naloxone	55.0 [33.2,76.8]	48.3 [41.4,55.2]		42 [39,45]
Other or unknown	0.0	0.0		13 [11,15]
**OAT median dose**	***n*** **= 20**	***n*** **= 195**		
Methadone	85 mg (min 45, max 160 mg)	75 mg (min 20, max 150 mg)	0.313	NA
Buprenorphine-naloxone	16 mg (min 10, max 20 mg)	16 mg (min 2, max 22 mg)	0.551	NA
**Years with HCV**	***n*** **= 1**	***n*** **= 107**		
median (min … max)	1	10 (min 0, max 29)		NA

*NA* Data not available

*HCV* Hepatitis C virus, *OAT* Opioid agonist therapy, *CI* Confidence interval.

^a^ Comparisons between the HCV treatment group and the other HCV antibody positive OAT patients, obtained from the Pearson’s chi-square test or the Mann-Whitney U test

^b^ The group ‘Other drugs’ includes benzodiazepines, gabapentinoids, cannabis, and/or other drugs typically taken orally

### Integrated HCV treatment: uptake and outcome

All the HCV-treated patients had filled out the PARADISE24fin questionnaire, which showed that the patients often experienced emotional difficulties, pain and sleeping problems (Table 2). However, the intensity and scope of the psychosocial difficulties experienced by the studied patients did not appear to differ from those identified in a larger group of OAT patients in Helsinki [19]. Overall, the OAT patients experienced somewhat less psychosocial difficulties than the inpatients with various substance use disorders (SUD) examined in the Addiction Hospital [20].

**Table 2:** The intensity and scope of psychosocial difficulties according to PARADISE24fin (scale: 0 = no difficulties/problems; 4 = extreme difficulties/cannot do)

Psychosocial difficulties	HCV-treated patients *n* = 20 Mean [95% CI]	OAT patients *n* = 269 [19] Mean [95% CI]	Inpatients with SUD *n* = 1082 [20] Mean [95% CI]
Difficulties in daily activities	0.9 [0.5,1.3]	0.8 [0.67,0.83]	1.2 [1.15,1.25]
Social difficulties	1.1 [0.7,1.5]	0.9 [0.80,0.98]	1.5 [1.45,1.55]
Cognitive difficulties	1.1 [0.7,1.5]	1.0 [0.90,1.11]	1.8 [1.73,1.87]
Difficulties in self-regulation	1.2 [0.9,1.6]	1.2 [1.06,1.24]	1.8 [1.75,1.85]
Emotional difficulties	1.6 [1.1,2.0]	1.5 [1.43,1.65]	2.2 [2.15,2.25]
Pain and sleeping problems	1.7 [1.3,2.1]	1.5 [1.40,1.62]	2.1 [2.02,2.18]
PARADISE24fin total mean	1.2 [0.9,1.6]	1.1 [1.02,1.18]	1.7 [1.66,1.74]

*HCV* Hepatitis C virus, *OAT* Opioid agonist therapy, *SUD* Substance use disorder, *CI* Confidence interval

Nineteen patients completed the HCV treatment and achieved an EOT SVR. Unfortunately, one patient was lost from the follow-up due to a non-treatment-related death. SVR12 was evaluated for 18 patients. One patient was lost from the follow-up before achieving SVR12. One patient who continued heavy intravenous drug use had relapsed or contracted a new infection by the time of evaluating SVR12. In total, 17 patients achieved SVR12.

The patients regarded extra psychosocial support during the HCV treatment as unnecessary. Due to a low level of participation, only three of the four psychosocial group meetings were finally arranged. No patients needed or requested for additional physician’s or nurse’s appointment.

Four patients seemed to somewhat improve their OAT performance during and after the HCV treatment. Two patients stopped their intravenous drug use because of the HCV treatment, and one of the patients stopped all concomitant drug use. One patient reported to have significantly cut down concomitant injecting drug use after the treatment, while still continuing to engage in sporadic use. These improvements were stable at the 1-year follow-up. None of the treated patients increased their concomitant drug use after completing the treatment.

### POC HCV testing

The majority of the patients had been screened for HCV using standard venous blood tests. Even though the laboratory personnel were experienced in collecting blood from patients with collapsed veins, only approximately 10% of the samples in our study could be collected with an evacuated blood collection system and the remaining samples were collected using butterfly needles. The volume of a blood sample that could be collected using any venous puncture method was too small in 3% of the patients willing to participate in the HCV laboratory testing.

Technical difficulties were not the only obstacles to HCV testing and treatment. Some OAT patients were unwilling to participate in any venous blood testing, which rendered them ineligible to partake in the HCV treatment. These patients reported that their main reason for refusing laboratory tests was a fear of difficulties with their veins and previous negative experiences in healthcare.

A total of 46 patients were screened both with standard venous blood test and dry blood spot testing (DBS), and 26 patients were screened only with POC testing using Xpert. The patients who were screened with DBS or POC testing preferred these methods to venous blood tests.

## Discussion

Of the identified HCV antibody positive OAT patients, 75% had positive HCV RNA, indicating a need for HCV treatment. Active concomitant drug use was common among the HCV antibody positive OAT patients and the 20 OAT patients who had already received HCV treatment. Active drug use among OAT HCV patients has also been reported elsewhere. For example, Norton et al. reported that among 36 OAT patients, 21 (58%) had active drug use [14]. In buprenorphine OAT, patients with HCV antibody have been significantly less likely to submit opioid-negative urinalysis, indicating a higher rate of concomitant opioid use [27].

Despite a high prevalence of concomitant drug use, extensive liver damage among HCV RNA positive OAT patients seemed to be somewhat rare. The majority of the patients screened with Fibroscan or APRI showed no significant liver damage. Based on these data, most HCV treatments could be implemented directly at a substance abuse treatment unit without the need to refer patients to specialized hospital care. The low rate of liver fibrosis could be linked to the rather short median time (10 years) since HCV diagnosis. According to a systematic review and meta-analysis of HCV progression in people who inject drugs, the average time to METAVIR stage F3 is 26–38 years, and to cirrhosis 34–46 years post-infection [28].

The 20 HCV-treated genotype 1 patients were rather similar to the other HCV positive OAT patients. Of the 20 patients receiving HCV treatment, 95% completed the 12-week treatment successfully as planned, and 85% achieved SVR12. This is in line with previous studies conducted among OAT patients, in which 65–100% have completed HCV DAA treatment, and 64–94% have achieved SVR [3]. A randomized clinical trial reported an SVR of 94% [15]. Another randomized clinical trial reported an SVR12 of 92% for OAT patients with HCV genotypes 1, 4, or 6 [16]. Higher SVR12 rates among OAT patients have also been reported: in one study, 100% of those with no active drug use and 95% of those with active drug use achieved SVR12 [14]. In our study, all patients with no drug use also achieved SVR12. The two patients achieving SVR but not SVR12 engaged in active, heavy concomitant injecting drug use.

Taking venous blood samples from OAT patients is known to be challenging. Due to negative previous experiences of primary healthcare and specialized hospital care services, some patients were reluctant to initiate testing. POC and DBS have proven effective in HCV testing, diagnostics, and treatment [24]. In our real-life setting, we found that special expertise and methods were often needed due to difficulties in taking HCV RNA samples from intravenous drug users who had collapsed veins. For this purpose, POC or DBS testing may lower the barrier of initiating testing and enable better compliance for patients with collapsed veins. While some of the POC methods demand purchasing expensive equipment, utilizing the dry blood spots (DBS) method enables an analysis of samples elsewhere and, additionally, does not require special sample storage or transportation, presumably further lowering the barriers for treatment evaluation.

Prior to large-scale HCV DAA treatments, it was unclear whether OAT patients were able to complete HCV treatments. Some of the reasons included modest outcomes and attachment to treatment with interferons, negative healthcare experiences and hesitation to focus treating patients with SUD [29, 30]. During recent years, many successful experiences have confirmed that patients on OAT have acquired SVR12, which our review further confirms. HCV treatments have been shown not be only feasible for patients on OAT, but also among needle and syringe exchange units, mobile services, and other settings [29]. Many of those studies have focused on the treatment of HCV specifically, without an effort to attach patients to care for treating their SUD, as funding mechanisms for SUD treatment differ in each country. Although OAT has been shown not to be a requirement for successful HCV testing and treatment, most OAT patients still require HCV treatment, and attaching patients to care has been shown to be beneficial for treating SUD in the long term [2, 3, 9, 10].

Despite the simplicity of HCV treatment with DAA, barriers to HCV treatment exist both among patients and providers [30]. Among patients, these barriers include current drug use and sobriety requirements to HCV treatment access, concerns about the side effects of the DAA therapy, stigma, gaps in continuity of care from diagnostics to treatment initiation, competing social responsibilities and mental health issues while facilitators included having a trustworthy provider and access to multidisciplinary HCV care [30]. Among providers, barriers to HCV treatment included lack of resources and lack of provider knowledge on HCV while facilitators included the simplicity of DAA therapy, co-location of HCV care with related health services, and professional identity as a doctor to advocate for patients [30]. We showed that many of these facilitators were available in an OAT setting. OAT patients’ own OAT addiction service unit naturally acted as a trustworthy provider facilitator, with no need for extra support as initially suggested by many payers. Even though the HCV-treated OAT patients had more current concomitant substance use than the average Finnish OAT patients, the integrated HCV treatment was simple for both the patients and the provider. The OAT patients in our review received their HCV treatment at two different OAT units. Based on the patients’ characteristics and psychosocial wellbeing, the studied OAT patients generally resembled other Finnish OAT patients but differed somewhat from inpatients at the Addiction hospital. Besides OAT, the integrated HCV testing and treatment protocol can be applied at other addiction service units, as well, if the aforementioned facilitators were present. This is important, especially, when the aim is to utilize the enforced attachment to care achieved with HCV treatment to further treat substance use disorders.

Our study is limited to using a retrospective approach and focusing on patients enrolled in a long-standing cohort. Standard patient records are not subject to the amount of quality checks typical for a research setting. The research data were partly incomplete and HCV treatment rates were low due to a lack of centralized funding for treatment and clinical evaluation, as many OAT clinics in Finland are run by private service providers and financed under municipal agreements. So far, HCV treatment rates have been generally low among OAT patients [9], and the treatment has been focused on the non-OAT, non-drug user population with mild fibrosis and conducted in specialized hospital care. The number of new infections has been higher than that of treated patients [8]. It is also possible that the first OAT patients who sought HCV treatment once all-oral DAA therapy became available without restrictions were those most motivated to start the treatment and commit to it. A major strength of this study is the detailed exploration of demographic and psychosocial factors associated with HCV treatment within OAT and a complete review of all available patient records.

## Conclusions

Based on the reviewed data, the liver function of the evaluated Finnish HCV positive OAT patients allows directly integrating HCV treatment into OAT addiction service units. Even though the number of the HCV-treated patients was low due to lack of financial commitment and doubts about the feasibility of treating HCV, the integrated HCV treatment model, consisting of performing all laboratory tests and providing the entire treatment and follow-up process at addiction service units, showed several potential benefits. Most of the patients in our study completed HCV treatment and achieved SVR12. Some patients seemed to have improved their adherence to OAT during and after the HCV treatment. In conclusion, integrating HCV testing and treatment into the Finnish OAT protocols produced promising results and warrants the treatment of OAT patients in larger numbers, with the possibility of facilitating the treatment of the addiction problem as a follow-up.

## Data Availability

The data underlying the study results cannot be shared publicly due to participant confidentiality. However, other researchers who provide a valid research question may request access to the data by submitting a proposal to the investigators. Any such proposals will be assessed by the national ethical review board.

## Abbreviations

ALT: Alanine aminotransferase
APRI: Aspartate aminotransferase to platelet ratio index
DAA: Direct-acting antiviral agent
DBS: Dry blood spot
EMA: European Medicines Agency
EMCDDA: European Monitoring Centre for Drugs and Drug Addiction
EOT: End of treatment
HCV: Hepatitis C virus
ICF: International Classification of Functioning, Disability and Health
IV: Intravenous
OAT: Opioid agonist therapy
POC: Point of care
RNA: Ribonucleic acid
SUD: Substance use disorder
SVR: Sustained virologic response
SVR12: Sustained virologic response at 12 weeks after the treatment
TDI: Treatment demand indicator
THL: Finnish Institute for Health and Welfare in Finland
